# Bilobalide Enhances AMPK Activity to Improve Liver Injury and Metabolic Disorders in STZ-Induced Diabetes in Immature Rats via Regulating HMGB1/TLR4/NF-*κ*B Signaling Pathway

**DOI:** 10.1155/2021/8835408

**Published:** 2021-04-17

**Authors:** Meng Zhao, Jianpin Qin, Wenting Shen, Aiping Wu

**Affiliations:** Department of Pediatrics, Xiangyang Central Hospital, Affiliated Hospital of Hubei University of Arts and Science, Xiangyang, 441021 Hubei Province, China

## Abstract

This study was aimed at examining the effect and underlying mechanisms of bilobalide (BB) on hepatic injury in streptozotocin- (STZ-) induced diabetes mellitus (DM) in immature rats. Immature rats (one day old) were randomly divided into five groups: group I, control nondiabetic rats; group II, STZ-induced, untreated diabetic rats; groups III/IV/V, STZ-induced and BB-treated diabetic rats, which were intraperitoneally injected with BB (2.5 mg/kg, 5 mg/kg, or 10 mg/kg) after 3 days followed by STZ treatment. We observed that BB improved the histopathological changes and maintained normal glucose metabolism, blood lipid, and liver function indicators, such as fasting blood glucose, obesity index, HbA1c, HOMA-IR, fast serum insulin, adiponectin, total cholesterol (TC), triglyceride (TG), high-density lipoprotein (HDL), low-density lipoprotein (LDL), aspartate transaminase (AST), and alanine transaminase (ALT) in STZ-induced DM in immature rats by a biochemical analyzer or ELISA. Meanwhile, Western blot analysis showed that in STZ-induced DM immature rats, BB decreased the expression of apoptosis-related proteins Bax, cleaved caspase-3, and cleaved caspase-9 while enhancing the Bcl-2 expression; BB downregulated the expression of ACC related to fat anabolism, while upregulating the expression of CPT-1 related to fat catabolism. Strikingly, treatment with BB significantly increased the expression of AMPK*α*1 as well as inhibited HMGB1, TLR4, and p-P65 expression in hepatic tissues of immature DM rats. AMPK inhibitor (compound C, CC) cotreated with BB undermined the protective effect of BB on the liver injury. The results of the present study suggested BB may have a significant role in alleviating liver damage in the STZ-induced immature DM rats.

## 1. Introduction

Diabetes mellitus (DM) is a metabolic disease characterized by hyperglycemia, which is a serious threat to human health [1]. The incidence of diabetes has been on the rise, with the global incidence of 6.4% in 2010 and projected to rise to 7.7% by 2030 [2]. And in China, the incidence of DM had increased to 11.6% by 2010 [3]. DM is a metabolic disorder of carbohydrates, proteins, and fats in the body caused by defects in insulin action and secretion. DM is associated with a high risk of cerebral thrombosis, cataract, cardiovascular, and nervous system disease [4]. Lipid metabolism disorders and increased lipid peroxidation contribute to the pathogenesis of the diabetic complication. Studies have confirmed that persistent hyperglycemia leads to abnormal biochemical indexes such as increased oxidative load in patients with DM, which eventually evolves into liver damage [5]. According to Scalone et al., DM costs an average of 3,315 euros per case per year, with 31.5% of the total drug cost [2]. Sulfonylurea drugs cause hypoglycemia, and a few patients will develop rashes and edema [6]. Biguanides cause weakness, vomiting, diarrhea, and lactic acidosis [7]. Glitazones cause edema, weight gain, and anemia [8]. Therefore, it is of great significance to develop new diabetes drugs.

Streptozotocin (STZ) is a naturally occurring nitrosourea, which has a selective destruction effect on the islet of certain species of animals and can induce insulin-dependent diabetes mellitus in many animals. In rat or mouse models, STZ given intravenously or intraperitoneally induces destruction of insulin-secreting *β* cells and eventually leads to diabetes [9, 10].

It is well known that ginkgo biloba extract (EGB) is a widely used botanical drug and it has a cytoprotective effect on hepatocytes. The report indicated that EGB significantly promoted antioxidant, antiproliferative, and proapoptotic effects in hepatoma cells [11]. The composition of EGB is complex, mainly including the active components of flavonoids and terpene lactones. Bilobalide (BB) is a sesquiterpene in EGB, accounting for 2.9%-3.2% [12]. Studies have shown that BB plays an important role in the treatment of cerebral ischemia, oxidative stress, and neuroprotective effects through regulating multiple signaling pathways. BB improved cerebral ischemia and reperfusion injury inhibition of proinflammatory mediator production and downregulation of JNK1/2 and p38 MAPK activation [13]. Meanwhile, BB protected BV2 microglia cells against OGD/reoxygenation injury by inhibiting TLR2/4 signaling pathways [14]. However, the role and mechanism of BB in the regulation of diabetes are still unclear. At present, traditional drugs for the treatment of diabetes are mainly insulin secretion and insulin sensitization, which will produce adverse reactions to patients, leading to a decline in patient compliance, leading to treatment failure [15]. Therefore, the purpose of this paper is to explore the protection of BB on diabetes in immature rats.

## 2. Materials and Methods

### 2.1. Animals and Model Construction

The immature type 1 DM rat model was constructed as per previous reports [16–18]. 66 healthy male Wistar rats (3~4 weeks old) were purchased from the Animal Experiment Center of Shandong University. To construct the immature type 1 DM rat model, newborn rats (one day old) were given a single intraperitoneal injection of freshly prepared streptozotocin (STZ, 50 mg/kg body weight, Sigma, St. Louis, MO, U.S.A.) in 0.1 mol/L citrate buffer (pH 4.5). As a comparison, adult Wistar rats (8 weeks old) were given a single intraperitoneal injection of freshly prepared streptozotocin (STZ, 50 mg/kg body weight, Sigma, St. Louis, MO, U.S.A.) in 0.1 mol/L citrate buffer (pH 4.5), too. The rats with blood glucose ≥ 16.8 mmol/L were considered as type 1 diabetic rats only after 96 hours after injection. The immature rats were placed with their own mothers for one month and kept in suitable temperature (22°C), humidity (70%), and 12 h light/dark in polyethylene cages. All experiments were conducted according to the 3R principle for animal experiments and approved by the ethics committee of the Affiliated Hospital of Hubei University of Arts and Science (No. SCXK (E) 2019–0004).

### 2.2. Experimental Protocol

Bilobalide (molecular forma: C_15_H_18_O_8_; molecular weight: 326.30; specification: ≥98%, 20 mg/vial; batch no.: A0166) was purchased from Chengdu Mansite Biotechnology Limited. 60 immature rats (one day old) and 35 adult rats (8 weeks old) were randomly divided into four parts. The first part (25 immature rats) was randomly divided into 5 groups (*n* = 5): healthy control, diabetes mellitus model (DM), and model administration groups (2.5 mg/kg, 5 mg/kg, and 10 mg/kg bilobalide). The control group was injected with 1% citrate-sodium citrate buffer and 40 mg/kg as placebo. According to the above method, the DM group rats were used to construct the model. After that, 1 mL of bilobalide was intraperitoneally injected once a day in each model administration group according to the dose. The rats were administered for 4 weeks. The second part was comprised of 25 adult rats and divided into the same groups and received the same treatments as the first group as an adult rat control. In the third part of rats (25 immature rats), the AMPK inhibitor test was performed. The rats were randomly divided into 5 groups: healthy control, diabetes mellitus model (DM), DM+bilobalide 10 mg/kg, DM+CC, and DM+bilobalide+CC. AMPK inhibitor dorsomorphin (compound C, CC) was purchased from Selleck, Inc., USA. 1 h after injecting intraperitoneally with CC (25 mg/kg) [19], the rats in the DM+CC group were modeled. 1 h after intraperitoneal injection of CC (25 mg/kg) and bilobalide (10 mg/kg), rat modeling was performed in the DM+bilobalide+CC group. The fourth part of rats (10 immature and 10 adults) was randomly divided into the healthy control group (*n* = 5) or the bilobalide group (10 mg/kg), to evaluate if bilobalide had influence on liver damage.

### 2.3. Western Blot Analysis

Hepatic tissues were lysed in a lysis buffer at 4°C for 30 min. Total protein was separated by 10% SDS polyacrylamide gel electrophoresis and then transferred to nitrocellulose membranes (Millipore, Boston, MA, USA). The membranes were blocked with 5% nonfat milk and incubated with corresponding protein antibodies or a rabbit anti-*β*-actin monoclonal antibody. Then, the membranes were subsequently incubated with a HRP goat anti-rabbit IgG (1 : 20000; Boster Bio, Wuhan, China; BA1054). The proteins were detected by a film scanner (Microtek, Shanghai, China), and *β*-actin was used as an internal control. The net optical density was analyzed with a gel image processing system (Image-Pro Plus 6.0). Primary antibodies used were as follows: Bax (Abcam, Cambridge, UK; cat. no. ab32503, 1 : 2000), Bcl-2 (CST, Boston, MA, USA; cat. no. 185002, 1 : 2000), caspase-3 (CST, Boston, MA, U.S.A.; cat. no.9662, 1 : 1000), caspase-9 (CST, Boston, MA, U.S.A.; cat. no.9504, 1 : 1000), ACC (CST, Boston, MA, U.S.A.; cat. no. 3662, 1 : 1000), CPT-1 (Abcam, Cambridge, UK; cat. no.189182, 1 : 2000), AMPK*α*1 (CST, Boston, MA, U.S.A.; cat. no. 2603, 1 : 1000), HMGB1 (Abcam, Cambridge, UK; cat. no. ab78923, 1 : 10000), TLR4 (Abcam, Cambridge, UK; cat. no. ab13556, 1 : 500), P65 (Abcam, Cambridge, UK; cat. no. ab16502, 1 : 2000), p-P65 (Abcam, Cambridge, UK; cat. no. ab53489, 1 : 1000), and *β*-actin (CST, Boston, MA, U.S.A.; cat. no. 4970, 1 : 1000).

### 2.4. Analytical Sample Preparation

After 4 weeks of treatment, the rats were deprived of food for 12 h and then anaesthetised by overexposure to CO_2_ gas. Blood samples were collected immediately, and serum was obtained by centrifugation (1,500 × g, 15 min, 4°C). After collecting the blood, liver samples were taken. Serum and liver samples were stored at -70°C for further analysis. Meanwhile, the liver of each rat was immediately excised. After washing with saline, sections of the liver were blotted dry and weighed, collected, and stored in liquid nitrogen until analysis.

### 2.5. Pathological Analysis of Liver Tissues

Liver samples were removed from the rats and fixed overnight with 40 gL^−1^ paraformaldehyde. Fixed tissues were embedded in paraffin, sliced into 3 *μ*m samples, and stained with haematoxylin and eosin (H&E). The stained areas were viewed using an optical microscope (Olympus CX31, Tokyo, Japan) with a magnifying power of ×100. The severity of liver injury was evaluated by Suzuki's criteria as previously reported [20]. In brief, no necrosis, congestion/centrilobular ballooning was given a score of 0, while severe congestion and more than 60% lobular necrosis were given a score of 4.

### 2.6. Detection of Experimental Indicators

The fasting glucose (FPG) levels of rats were measured by a microglycemeter and test paper (Eppendorf, Germany). An automatic biochemical analyzer (Hitachi, Japan) detected blood lipid indicators: total cholesterol (TC), triglyceride (TG), high-density lipoprotein (HDL), low-density lipoprotein (LDL), alanine transaminase (ALT), and aspartate transaminase (AST).

### 2.7. Apoptosis Assay

The apoptosis of liver tissues was assessed by transferase-mediated dUTP nick end labeling (TUNEL) apoptosis detection kit (Abbkine Scientific Co., Ltd., California, USA) according to the manufacturer's instructions.

### 2.8. Oil Red O Staining

The sections were washed with PBS 3 times, fixed with 4% paraformaldehyde solution for 10 min, washed with PBS 3 times, rinsed with 60% isopropyl alcohol for 10 sec, and added with 0.3% oil red O staining solution for 1 min. After being rinsed with 60% isopropyl alcohol and being rinsed with PBS 3 times, the sections were observed and photographed under a microscope (Olympus, Tokyo, Japan).

### 2.9. Statistical Analysis

In this study, all experiments were performed in triplicate at a minimum. Results are expressed as mean ± deviation (mean ± SD) and analyzed by one-way ANOVA and LSD (least significant difference) test using SPSS software version 22.0 (SPSS, Inc., Chicago, IL, USA). Differences were considered statistically significant at *P* value < 0.05.

## 3. Results

### 3.1. Effect of BB on the Liver Damage and Glucose Metabolism in STZ-Induced Immature Diabetic Rats

**Figure 1:**
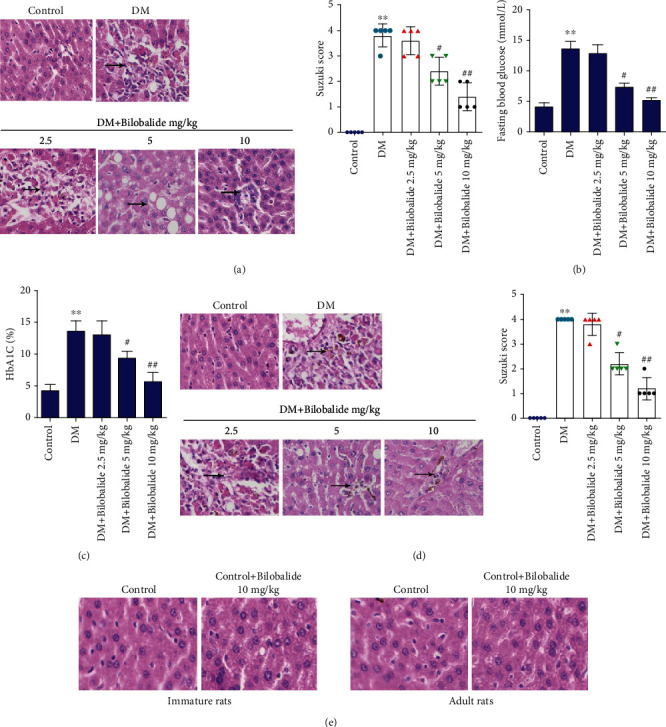
Effect of bilobalide (BB) supplementation on the liver damage and glucose metabolism in STZ-induced diabetic rats. The one-day-old immature rats or eight-week-old adult rats were randomly allocated to 5 groups (*n* = 5–6/group): healthy control, diabetes mellitus model (DM), and drug treatment groups (the rats were intraperitoneally injected with either low (2.5 mg/kg), medium (5 mg/kg), or high (10 mg/kg) dose of bilobalide). (a) The degree of liver damage in immature rats was identified by haematoxylin and eosin (H&E) staining and Suzuki score. (b, c) Glucose and HbA1c levels were determined using commercial kits. (d) The degree of liver damage in adult rats was identified by haematoxylin and eosin (H&E) staining and Suzuki score. (e) The degree of liver damage in immature and adult rats treated with 10 mg/kg bilobalide was identified by haematoxylin and eosin (H&E) staining. ^∗∗^*P* < 0.01 (vs. control); ^#^*P* < 0.05 (vs. DM); ^##^*P* < 0.01 (vs. DM).

Compared with the control group, the STZ-induced diabetic immature rats showed significant liver damage with increasing Suzuki score (Figure 1(a)). The BB treatment (5 mg/kg and 10 mg/kg) dose-dependently ameliorated the change as compared to the DM group, with decreasing Suzuki score (Figure 1(a)). After injection of STZ in immature rats, fasting blood glucose levels and HbA1c levels were significantly increased (Figures 1(b) and 1(c)). The administration of BB (5 mg/kg and 10 mg/kg) resulted in a significant reduction of glucose and HbA1c levels as compared to the DM group (Figures 1(b) and 1(c)). In adult rats, STZ-induced type 1 DM also significantly increased the liver damage with an increasing Suzuki score compared with the control group, while BB treatment dose-dependently reduced liver damage with a decreasing Suzuki score (Figure 1(d)). Moreover, we should notice that BB treatment (10 mg/kg) did not cause liver damage in normal rats (both immature and adult), indicating that the doses of BB we used in our study were safe without apparent cytotoxicity (Figure 1(e)).

### 3.2. Effect of BB on the Liver Biochemical Indicators in STZ-Induced Immature Diabetic Rats

**Figure 2:**
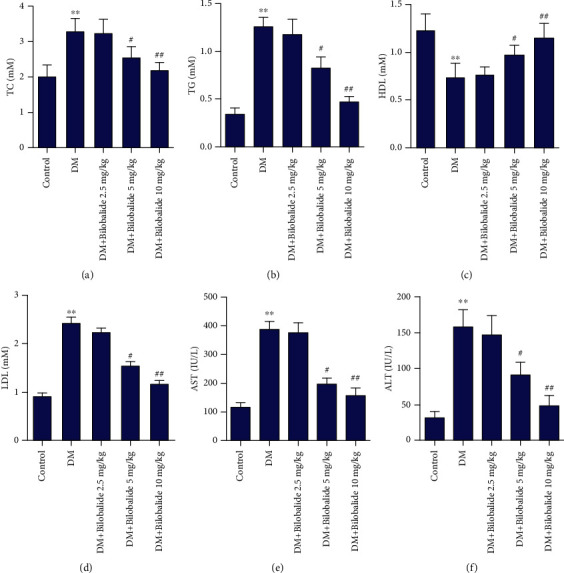
Effect of BB supplementation on the liver biochemical indicators in STZ-induced diabetic rats. Total cholesterol (TC), triglyceride (TG), high-density lipoprotein (HDL), low-density lipoprotein (LDL), alanine transaminase (ALT), and aspartate transaminase (AST) levels were determined using commercial kits. ^∗∗^*P* < 0.01 (vs. control); ^#^*P* < 0.05 (vs. DM); ^##^*P* < 0.01 (vs. DM).

TC and TG levels were significantly increased in STZ-treated immature rats as compared to the control value (Figures 2(a) and 2(b)). The coadministration of BB (5 mg/kg and 10 mg/kg) with STZ resulted in significant reduction in TC and TG levels as compared to the DM group (Figures 2(a) and 2(b)). Furthermore, concentration of HDL was markedly decreased while that of LDL was increased after STZ injection compared to the control group (Figures 2(c) and 2(d)). Administration of BB (5 mg/kg and 10 mg/kg) followed by STZ injection significantly activated HDL levels and weakened LDL levels dose-dependently (Figures 2(c) and 2(d)). Strikingly, STZ administration was associated with highly significant increases in AST and ALT levels as shown in Figures 2(e) and 2(f). Injection of BB (5 mg/kg and 10 mg/kg) following STZ administration dose-dependently inhibited these elevations (Figures 2(e) and 2(f)).

### 3.3. Effect of BB on Liver Cell Apoptosis in STZ-Induced Immature Diabetic Rats

**Figure 3:**
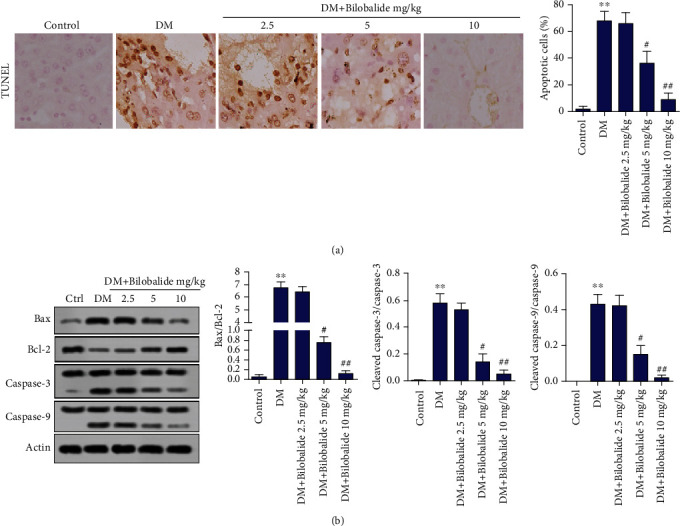
Effect of BB supplementation on liver cell apoptosis in STZ-induced diabetic rats. (a) The apoptosis of liver cells was detected by TUNEL assay. (b) The expressions of Bax, Bcl-2, caspase-3, cleaved caspase-3, caspase-9, and cleaved caspase-9 in hepatic tissues were assayed using Western blot analysis. *β*-Actin is a loading control. ^∗∗^*P* < 0.01 (vs. control); ^#^*P* < 0.05 (vs. DM); ^##^*P* < 0.01 (vs. DM).

As shown in Figure 3(a), the liver cell apoptosis in the DM group was increased compared with that in the control group. Administration of BB (5 mg/kg and 10 mg/kg) together with STZ inhibited the apoptosis level as compared to the STZ group (Figure 3(a)). Moreover, Western blot analysis showed that the expressions of apoptosis-related proteins Bax, cleaved caspase-3, and cleaved caspase-9 were enhanced while Bcl-2 expression was decreased in the DM group (Figure 3(b)). BB (5 mg/kg and 10 mg/kg) treatment in combination with STZ dose-dependently hindered these increased levels compared with rats in the DM group (Figure 3(b)).

### 3.4. Effect of BB on Lipid Metabolism in STZ-Induced Immature Diabetic Rats

**Figure 4:**
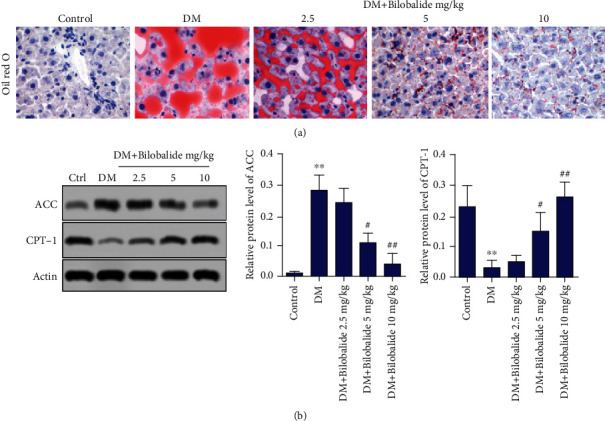
Effect of BB supplementation on lipid metabolism in STZ-induced diabetic rats. (a) The degree of liver fat accumulation was detected by oil red O staining. (b) The expressions of acetyl-CoA carboxylase (ACC) and carnitine palmitoyltransferase 1 (CPT-1) in hepatic tissues were assayed using Western blot analysis. *β*-Actin is a loading control. ^∗∗^*P* < 0.01 (vs. control); ^#^*P* < 0.05 (vs. DM); ^##^*P* < 0.01 (vs. DM).

Oil red O staining showed there was more severe accumulation of fat in STZ-induced diabetic rats than the control group (Figure 4(a)). BB (5 mg/kg and 10 mg/kg) treatment in combination with STZ dose-dependently reduced fat accumulation as compared with untreated diabetic rats (Figure 4(a)). In addition, it is well known that acetyl-CoA carboxylase (ACC) and carnitine palmitoyltransferase 1 (CPT-1) have important roles in the control of fat oxidation [21, 22]. We observed the obvious increase in the ACC expression and the decrease in CPT-1 expression in hepatic tissues of DM rats compared with the control group (Figure 4(b)). Administration of BB (5 mg/kg and 10 mg/kg) dose-dependently weakened the expression of ACC while strengthening the levels of CPT-1 compared with the DM group (Figure 4(b)).

### 3.5. BB Modulated Hepatic Injury in STZ-Induced Immature Diabetic Rats through AMPK Activation and HMGB1/TLR4/NF-*κ*B Signaling Pathway

**Figure 5:**
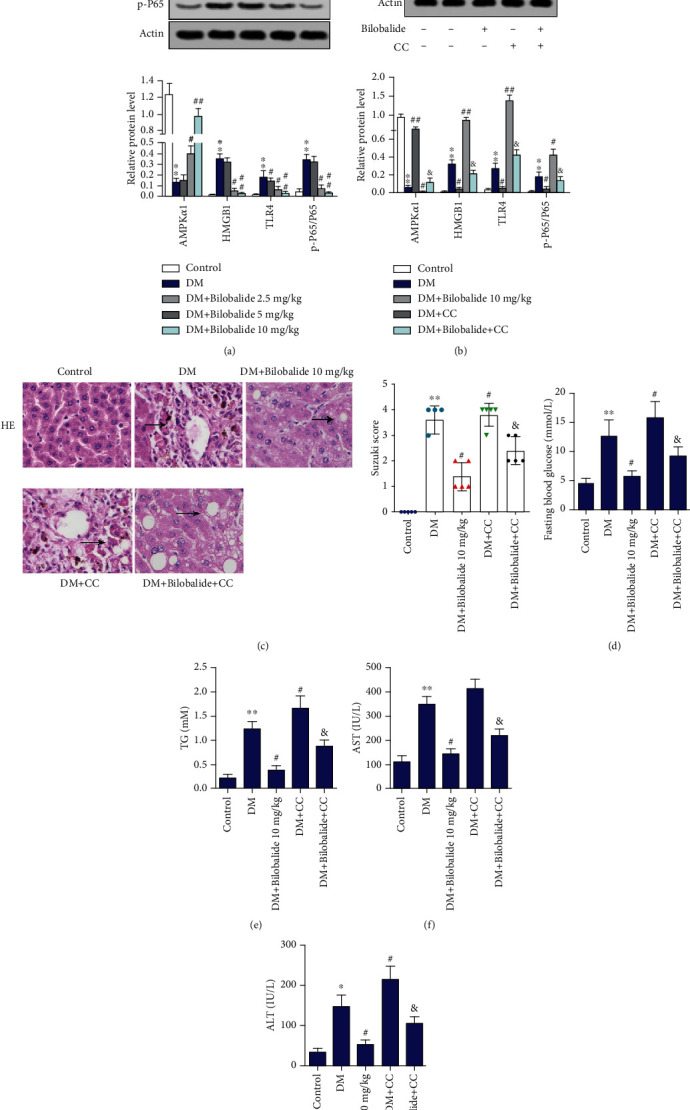
BB modulated hepatic antioxidant in STZ-induced diabetic rats through AMPK activation and HMGB1/TLR4/NF-*κ*B signaling pathway. The one-day-old immature rats were randomly allocated to 5 groups: healthy control, diabetes mellitus model (DM), STZ+BB (10 mg/kg), STZ+AMPK inhibitor (compound C, CC), STZ+BB (10 mg/kg)+CC. (a, b) The expressions of AMPK*α*1, HMGB1, TLR4, P65, and p-P65 in hepatic tissues were assayed using Western blot analysis. *β*-Actin is a loading control. (c) The degree of liver damage was identified by H&E staining and Suzuki score. (d–g) Glucose, TG, AST, and ALT levels were determined using commercial kits. ^∗∗^*P* < 0.01 (vs. control); ^#^*P* < 0.05 (vs. DM); ^##^*P* < 0.01 (vs. DM); ^&^*P* < 0.05 (vs. DM+BB (10 mg/kg)).

Studies have shown that AMPK activation suppressed de novo lipogenesis, and the HMGB1/TLR4/NF-*κ*B pathway was involved in liver inflammation [23, 24]. Thus, we further investigated whether BB can regulate the AMPK and HMGB1/TLR4/NF-*κ*B expression in STZ-induced diabetic rats. Our results suggested that AMPK*α*1 expression was blocked, as well as that HMGB1, TLR4, and p-P65 expression was increased in STZ-induced diabetic rats (Figure 5(a)). And BB treatment (5 mg/kg and 10 mg/kg) dose-dependently activated the AMPK*α*1 expression and weakened the HMGB1, TLR4, and p-P65 expression compared with the model group (Figure 5(a)). Thus, the high dose of BB (10 mg/kg) was used for subsequent experiments. Wistar rats were randomly allocated to 5 groups: healthy control, diabetes mellitus model (DM), STZ+BB (10 mg/kg), STZ+AMPK inhibitor (compound C, CC), and STZ+BB (10 mg/kg)+CC. Western blot analysis proved that CC administration inhibited AMPK*α*1 expression and inspired HMGB1, TLR4, and p-P65 expression compared with the BB treatment group (Figure 5(b)). Notably, inhibition of AMPK obviously deteriorated liver damage compared with the BB-injected group, while increasing the Suzuki score (Figure 5(c)). Significant increases of glucose, TG, AST, and ALT levels were observed in rats treated with CC compared with the BB-injected group (Figures 5(d)–5(g)).

## 4. Discussion

This study was designed to evaluate the effects and mechanisms of BB on improving hepatic injury in STZ-induced diabetes in immature rats. The immature rats treated with STZ on the first day of birth exhibited deficiency of insulin secretion and an increase of HbA1c level. Moreover, the immature rats treated with STZ showed obvious liver damage compared with the control group, and this was corresponding with previous studies [16, 18]. Shinde and Goyal found that inducing of type 1 DM in neonatal rats caused apparent liver damage with increase in intensity and incidence of vacuolations [16]. Jaiswal et al. also found that the immature diabetic rats developed severe liver injury at 4 weeks after STZ injection [18]. In our study, we found that BB treatment obviously alleviated liver damage caused by type 1 DM in neonatal rats. The results of this study showed significant effects of BB on reducing the blood glucose concentration and activating insulin secretion.

High concentration of glucose has been shown to be associated with liver oxidative damage in DM [25, 26]. Insulin deficiency causes metabolic disorders and the accumulation of harmful substances in the liver leading to severe oxidative stress [27]. STZ treatment to rats leads to liver biochemical disorders, which is manifested by the abnormal expression of active substances, such as ALT and AST [28]. The results of this study clearly showed the high level of ALT and AST serums in STZ-induced immature diabetic rats. In contrast, BB caused a significant decrease in the levels of ALT and AST. On the other hand, the liver is generally known to be the central regulator of lipid homoeostasis. Many studies have shown that elevated levels of plasma free fatty acids in DM patients stimulate the liver to synthesize and secret large amounts of TG and LDL [28–30]. In our present study, we found that the levels of TC, TG, LDL, and HDL were abnormal in STZ-induced diabetic rats. And BB improved liver function as reflected by the levels of biomarkers mentioned above. Consistently, oil red O staining indicated that BB inhibits lipid accumulation in the hepatic tissue. Mechanistically, BB treatment improved lipid metabolic balance in the liver of immature DM rats by reducing the fatty acid oxidation marker enzyme ACC expression and enhancing CPT-1 activity. The results are in agreement with those reported in earlier studies which found that hyperglycemia activated ACC, hence increasing malonyl-CoA and reducing CPT-1 activity [31, 32]. Growing evidences showed that the process of cell death by apoptosis was a ubiquitous phenomenon in hepatic cell injury. STZ-mediated hyperglycemia obviously enhanced mitochondrial Bax expression, cytosolic cytochrome C levels, and caspase-3 activity leading to an increase in the apoptotic index [33]. Additionally, apoptotic protein cleaved caspase-3 was increased and antiapoptotic signaling protein B-cell lymphoma 2 (Bcl-2) was decreased in DM rats [34]. Our in vivo studies demonstrated that STZ led to increases of Bax, cleaved caspase-3, and cleaved caspase-9 as well as a decrease of Bcl-2 expression in rat liver, which was significantly reduced by BB treatment. In order to clarify BB hepatic damage in STZ-induced DM rats, we revealed the molecular mechanisms in the present study.

AMPK activation has been shown to enhance glucose entry into cells and inhibit intracellular glucose production. However, AMPK activity was impaired in diabetic patients. Natural products have shown significant potential in modulating and activating the AMPK pathway, which controls diabetes and its complications [35]. It has been reported that the expressions of fatty triglyceride lipase, hormone sensitive lipase (HSL), and carnitine palmitoyltransferase 1 are upregulated under the action of bilobalide, and the phosphorylation of AMP activated protein kinase (AMPK), acetyl-CoA carboxylase 1, and HSL is stimulated. In addition, bilobalide partially restored AMPK activity after compound C (dorsomorphin) blocked the AMPK activity. These results suggest that dibromide inhibits adipogenesis in 3T3-L1 cells and promotes adipolysis by activating AMPK signaling pathways [36]. In this study, the BB treatment had a significant effect on activation AMPK*α*1. Researchers showed that HMGB1 and TLR4 were reported to be closely related to inflammation and liver failure [37, 38]. HMGB1 could activate NF-*κ*B through TLR4 and receptor for advanced glycation end products (RAGEs), resulting in the development of inflammatory response [39]. Notably, TLR4 and lipopolysaccharide (LPS) interaction is closely related to liver injury via the mediating immune signaling pathway [40]. Studies have shown that bilobalide protects BV2 microglia from OGD/reoxygenation damage by inhibiting the TLR2/4 signaling pathway [14]. In our study, we found that the levels of HMGB1, TLR4, and NF-*κ*B (p-P65) expression in the hepatic tissues of STZ-induced immature DM rats were higher than those of the control rats. And BB intervention significantly reversed these alterations.

In our study, we also found that BB treatment alleviated liver damage caused by STZ-induced diabetes in adult rats, which was similar with that in immature rats. However, we chose to conduct our experiments in neonatal rats because we aimed to explore the influence of BB on type 1 DM of young individuals. There are also limitations for our STZ-induced type 1 DM immature rat model. Type 1 DM is characterized by selective autoimmune destruction of the pancreatic beta cells, and STZ shows specific toxicity to pancreatic beta cells. Previous studies indicate that animals with STZ-induced type 1 DM exhibit changed immune responses [41–43]. However, the immune responses of STZ-induced type 1 DM in neonatal rats were undetermined in our study. Whether our immature rat model could produce immunoresistance to insulin is unknown. There is possibility for our animal model to produce immunoresistance to insulin, but this is to be determined. As our study mainly focused on the function of BB in neonatal DM rats, our STZ-induced type 1 DM immature rat model was sufficient for exploring this.

In summary, the results presented here showed that administration of BB activated the expression of AMPK*α*1 as well as inhibited HMGB1, TLR4, and p-P65 expression in the liver of STZ-induced immature DM rats. We believe that BB might be considered as a potential adjuvant entity for improving hepatic injury in young DM patients.

## Data Availability

The data used to support the findings of this study are available from the corresponding author upon request.
